# Target Localization in Wireless Sensor Networks Based on Received Signal Strength and Convex Relaxation

**DOI:** 10.3390/s22030733

**Published:** 2022-01-19

**Authors:** Weizhong Ding, Qiubo Zhong, Yan Wang, Chao Guan, Baofu Fang

**Affiliations:** Robotics Institute, Ningbo University of Technology, Ningbo 315048, China; weizhongdingnubt@163.com (W.D.); wangy0201@nbut.edu.cn (Y.W.); c.guan@nbut.edu.cn (C.G.); fangbf@hfut.edu.cn (B.F.)

**Keywords:** target localization, maximum likelihood estimation, received signal strength, semi-definite programming, wireless sensor networks

## Abstract

A new positioning algorithm based on RSS measurement is proposed. The algorithm adopts maximum likelihood estimation and semi-definite programming. The received signal strength model is transformed to a non-convex estimator for the positioning of the target using the maximum likelihood estimation. The non-convex estimator is then transformed into a convex estimator by semi-definite programming, and the global minimum of the target location estimation is obtained. This algorithm aims at the $L_{0}$ known problem and then extends its application to the case of $L_{0}$ unknown. The simulations and experimental results show that the proposed algorithm has better accuracy than the existing positioning algorithms.

## 1. Introduction

With the development of wireless sensor networks (WSNs) [1,2,3,4,5,6,7,8,9,10,11,12,13], the Internet of things has become common. Positioning algorithms are widely used in intelligent warehouse, robot cooperation, instrument navigation, position monitoring, etc. The predefined points are known as the anchors, while the target refers to the point whose position needs to be estimated. The common measurements are angle of arrival (AOA) [14,15,16,17], time of arrival (TOA) [18,19,20], time difference of arrival (TDOA) [21,22] and received signal strength (RSS) [23,24,25,26,27,28,29]. However, AOA requires antenna arrays. TOA and TDOA require clock synchronization, which greatly increases the costs. Compared with other measurements, the RSS measurement is easier and a lower cost to obtain. Hence, we focus on it in this paper. There are two models of RSS: one is based on signal strength, and the other is on path loss. This paper adopts the latter.

Among the existing RSS positioning algorithms, a common approach is to construct a non-convex function about the target and then find the extreme value of the function using some mathematical algorithms, such as gradient descent, coordinate descent, genetic algorithm, and golden section algorithm. However, because of the noise in the received signal, the extremum of the non-convex equation may not be the global optimal solution, but the local optimum instead. Therefore, a more accepted approach is using convex optimization schemes to get a convex equation, and then find the global optimal value. The least squares (LS), least relative error (LSRE) and weighted least squares (WLS) are applied in most optimization problems. In the literature, Robin, WO. [30], Tomic, S. [31], Wang, Z. [32], Surya, VP. [33] and Mei, X. [34], all choose to ignore the difference of the standard deviation of noise after the Taylor expansion of the term containing the amount of noise in the model transformation. Therefore, if the received signal strength contains noise, the final estimated position has some deviation, which increases as the noise standard deviation increases, and the deviation also increases, resulting in the poor estimation accuracy. The SOCP1 method proposed by Tomic, S. [35] and the SDP1 method proposed by Chang, S. [36] used the convex optimization to solve this problem. They also considered cooperative scenes. Coluccia, A. proposed a Bayesian formulation of the ranging problem [37]. This method is called “optimal ML range-free”. The DEOR method [38] and DEOR-fast method [39] proposed by Najarro, L.A.C. employed the three techniques of DE, OBL and adaptive redirection. These two methods achieved good accuracy, but the lower and upper bounds for the initial population is hard to be sure about. This paper proposes a new positioning algorithm based on maximum likelihood estimation and semi-definite programming (“MLE-SDP”), which takes into account the variation of noise standard deviation. The basic steps of this algorithm is to transform the path loss model of the received signal into a relatively simple expression without a logarithm and expand the term with noise by Taylor series to obtain a new noise term. The variance of the new term is proportional to the distance between the target and each anchor. Then, the maximum likelihood estimation function is constructed, and a non-convex estimator is obtained. Next, we use semi-definite programming [40] to transform the non-convex variables and obtain a constrained convex estimate. The estimator of the target can be obtained by solving the convex problem.

The main contributions of this paper is as follows:

1. The RSS model is transformed into a pseudo-linear system with new noise;
2. Based on LS criterion, a new non-convex objective function is derived to solve the target positioning problem;
3. The non-convex objective function is transformed into a convex objective function by semi-definite programming.

The experiments were carried to verify the performance of the proposed algorithm. It is compared with three existing common algorithms and the Cramer–Rao lower bound (CRLB). The simulation results show that the proposed algorithm achieves substantial improvements in accuracy, at the same complexity and running time. The results of field test are also given in Section 5.

## 2. System Model and Problem Formulation

**Figure 1:**
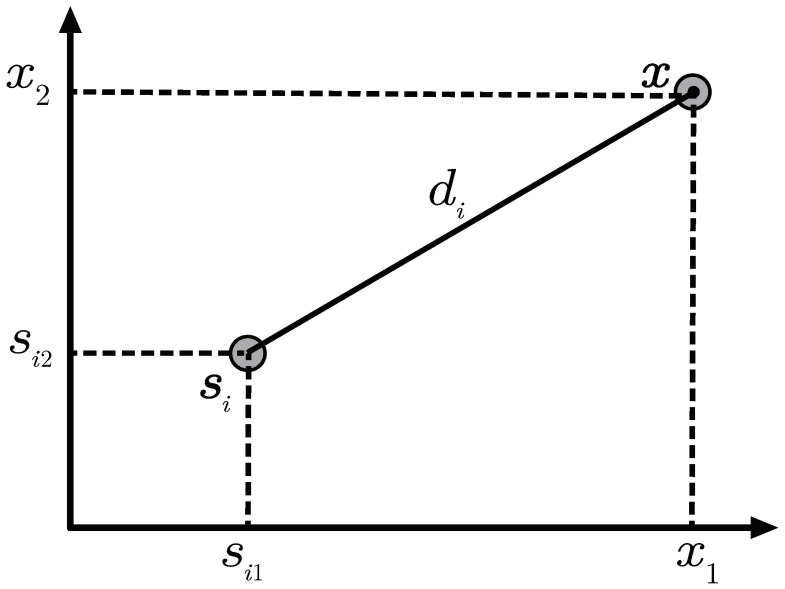
Schematic diagram of anchor and target position.

Suppose a two-dimensional (or three-dimensional) sensor network is composed of *N* anchors and a target with unknown position. The positions of the target and the *i*-th anchor are represented by x and $s_{i}$ (i=1,…,N), respectively. As show in Figure 1, $d_{i}$ represent the distance between the i-th anchor and the target. Assuming that the received signal noise follows the normal distribution $n_{i}∼N\left(0,\sigma_{n_{i}}^{2}\right)$. That means the mean of $n_{i}$ is zero and the standard deviation is $\sigma_{n_{i}}$, making the system model to be

(1) $$\begin{array}{c} L_{i}=L_{0}+10\gamma \log_{10}\frac{∥x-s_{i}∥}{d_{0}}+n_{i},\;i=1,\ldots ,N \end{array}$$

where $L_{i}$ is the RSS measurement received by the i-th anchor, and $L_{0}$ is the RSS measurement received by the anchor. When the distance between the anchor and the target is $d_{0}$, $d_{0}$ is the reference distance, usually set as 1 m. γ is the path loss index, and it is generally between 2.2 and 4. This paper takes 2.2 because this paper is based on LOS scenes. $n_{i}$ is the received signal noise of the *i*-th measurement. When $L_{0}$ is known, the maximum likelihood estimation is used as

(2) $$\begin{array}{c} \min_{x}\;\sum_{i=1}^{N}\frac{{\left(L_{i}-L_{0}-10\gamma \log_{10}\frac{∥x-s_{i}∥}{d_{0}}\right)}^{2}}{{\sigma_{n_{i}}}^{2}},\;i=1,\ldots ,N. \end{array}$$

When $L_{0}$ is unknown, the maximum likelihood estimation is used as

(3) $$\begin{array}{c} \min_{x,L_{0}}\;\sum_{i=1}^{N}\frac{{\left(L_{i}-L_{0}-10\gamma \log_{10}\frac{∥x-s_{i}∥}{d_{0}}\right)}^{2}}{{\sigma_{n_{i}}}^{2}},\;i=1,\ldots ,N. \end{array}$$

Because large-scale examples of these problems cannot be solved by accurate algorithm, Equations (2) and (3) are NP-hard problems which are very complex non-convex estimators, and it is difficult to find the global optimal solution.

## 3. The Proposed Algorithm

In this section, we introduce the estimator of the target position based on the maximum likelihood estimation in the two-dimensional case and use semi-definite programming to change the non-convex estimator into convex, so that we can find the global optimal solution. For 3D, it is similar to 2D.

In Section 3.1, $L_{0}$ is known, and in Section 3.2, $L_{0}$ is unknown. Table 1 lists the symbols that appear frequently in this section.

### 3.1. $L_{0}$ Known Positioning Algorithm

To simplify the system model, subtracting $L_{0}$ from both sides of Equation (1) and dividing by 10γ, and taking the power of 10 at the same time, we obtain

(4) $$\begin{array}{c} d_{0}10^{\frac{L_{i}-L_{0}}{10\gamma }}=∥x-s_{i}∥10^{\frac{n_{i}}{10\gamma }},\;i=1,\ldots ,N. \end{array}$$

Let us expand $10^{\frac{n_{i}}{10\gamma }}$ by McLaughlin, just $10^{\frac{n_{i}}{10\gamma }}\approx 1+\frac{\ln10}{10\gamma }n_{i}$, and we obtain

(5) $$\begin{array}{c} \alpha_{i}\eta =∥x-s_{i}∥+\frac{\ln10}{10\gamma }∥x-s_{i}∥n_{i},\;i=1,\ldots ,N \end{array}$$

where $\alpha_{i}=d_{0}10^{\frac{L_{i}}{10\gamma }}$, $\eta =10^{-\frac{L_{0}}{10\gamma }}$. According to $\alpha_{i}\eta \approx ∥x-s_{i}∥$, we obtain

(6) $$\begin{array}{c} \alpha_{i}\eta =∥x-s_{i}∥+\xi_{i},\;i=1,\ldots ,N \end{array}$$

where $\xi_{i}=\frac{\ln10}{10\gamma }\alpha_{i}\eta n_{i}$, a new noise $\xi_{i}$ with mean square error $\frac{\ln10}{10\gamma }\alpha_{i}\eta \sigma$ is obtained, a non-convex equation is constructed according to maximum likelihood estimation as

(7) $$\begin{array}{c} \min_{x}\;\underset{i=1}{\sum^{N}}\;\frac{{\left(\alpha_{i}\eta -∥x-s_{i}∥\right)}^{2}}{{\left(\frac{\ln10}{10\gamma }\alpha_{i}\eta \sigma \right)}^{2}} \end{array}$$

because $\frac{\ln10}{10\gamma }$, η, σ are constant, they are not affected when estimating x. Thus, they can be removed, an unfolding molecule, and we obtain

(8) $$\begin{array}{c} \min_{x}\;\underset{i=1}{\sum^{N}}\;\frac{{∥x-s_{i}∥}^{2}-2\alpha_{i}\eta ∥x-s_{i}∥}{{\alpha_{i}}^{2}}+1 \end{array}$$

The addition of 1 in the objective function does not affect the estimated value x. Thus, Equation (8) can be written as

(9a) $$\begin{array}{c} \min_{x,d,r}\;\underset{i=1}{\sum^{N}}\;\frac{r_{i}-2\alpha_{i}\eta d_{i}}{{\alpha_{i}}^{2}} \end{array}$$

(9b) $$\begin{array}{c} \;s.t.\;d_{i}=∥x-s_{i}∥ \end{array}$$

(9c) $$\begin{array}{c} r_{i}={d_{i}}^{2} \end{array}$$

where $d_{i}=∥x-s_{i}∥$, $r_{i}={d_{i}}^{2}$. Now, the objective function is transformed into convex, and then the constraints are transformed as

(10a) $$\begin{array}{c} \min_{x,y,d,r}\;\;\underset{i=1}{\sum^{N}}\;\frac{r_{i}-2\alpha_{i}\eta d_{i}}{{\alpha_{i}}^{2}} \end{array}$$

(10b) $$\begin{array}{c} s.t.\;r_{i}=y-2{s_{i}}^{T}x+{s_{i}}^{T}s_{i} \end{array}$$

(10c) $$\begin{array}{c} y=x^{T}x \end{array}$$

(10d) $$\begin{array}{c} r_{i}={d_{i}}^{2}. \end{array}$$

Using semi-definite programming, we transform Equation (10) into convex and obtain the final estimator as

(11a) $$\begin{array}{c} \min_{x,y,d,r}\;\;\underset{i=1}{\sum^{N}}\;\frac{r_{i}-2\alpha_{i}\eta d_{i}}{{\alpha_{i}}^{2}} \end{array}$$

(11b) $$\begin{array}{c} \;s.t.\;r_{i}=y-2{s_{i}}^{T}x+{s_{i}}^{T}s_{i} \end{array}$$

(11c) $$\begin{array}{c} \left[\begin{array}{cc} x^{T} & y\\ I_{2} & x \end{array}\right]\geq 0 \end{array}$$

(11d) $$\begin{array}{c} \left[\begin{array}{cc} d_{i} & r_{i}\\ 1 & d_{i}. \end{array}\right]\geq 0 \end{array}$$

The estimator Equation (11) is a new positioning algorithm when $L_{0}$ is known, and it is called “MLE-SDP”.

For Equation (10), the form of second-order cone programming can also be used, and the transformed estimator Equation (12) is called “MLE-SOCP” as

(12a) $$\begin{array}{cc} \; & \min_{x,y,d,r}\;\;\underset{i=1}{\sum^{N}}\;\frac{r_{i}-2\alpha_{i}\eta d_{i}}{{\alpha_{i}}^{2}} \end{array}$$

(12b) $$\begin{array}{cc} \;s.t.\;r_{i} & =y-2{s_{i}}^{T}x+{s_{i}}^{T}s_{i} \end{array}$$

(12c) $$\begin{array}{cc} \; & ∥\left[\begin{array}{c} 2x\\ 1-y \end{array}\right]∥\leq 1+y \end{array}$$

(12d) $$\begin{array}{cc} \; & ∥\left[\begin{array}{c} 2d_{i}\\ 1-r_{i}. \end{array}\right]∥\leq 1+r_{i} \end{array}$$

Because the simulation results of the “MLE-SDP” algorithm are better than the “MLE-SOCP” algorithm’s, this paper only uses the “MLE-SDP” algorithm to compare with other algorithms.

### 3.2. $L_{0}$ Unknown Positioning Algorithm

Because the transformation of $L_{0}$ unknown case is very similar to $L_{0}$ known case, the final estimator is given directly as

(13a) $$\begin{array}{c} \min_{x,y,d,r,\eta ,u}\;\;\underset{i=1}{\sum^{N}}\;\frac{r_{i}-2\alpha_{i}u_{i}}{{\alpha_{i}}^{2}} \end{array}$$

(13b) $$\begin{array}{c} \;s.t.\;r_{i}=y-2{s_{i}}^{T}x+{s_{i}}^{T}s_{i} \end{array}$$

(13c) $$\begin{array}{c} \left[\begin{array}{cc} x^{T} & y\\ I_{2} & x \end{array}\right]\geq 0 \end{array}$$

(13d) $$\begin{array}{c} \left[\begin{array}{cc} d_{i} & r_{i}\\ 1 & d_{i} \end{array}\right]\geq 0 \end{array}$$

(13e) $$\begin{array}{c} \left[\begin{array}{cc} \eta & u_{i}\\ 1 & d_{i} \end{array}\right]\geq 0 \end{array}$$

the estimator Equation (13) is the positioning algorithm, when $L_{0}$ is unknown, called “MLE-SDP2” in this paper.

## 4. Simulation Results

In this section, the performance of the proposed algorithms “MLE-SDP” and “MLE-SDP2” are verified by MATLAB simulations. In Section 4.1, the “MLE-SDP” algorithm is compared with the “LS-SDP” algorithm, the “LS-SOCP” algorithm, the “LSRE-SOCP” algorithm, the “optimal ML range-free” algorithm, the “SOCP1” algorithm, the “DEOR1” algorithm and the “DEOR-fast1” algorithm. In Section 4.2, the “MLE-SDP2” algorithm is compared with the “LS-SDP2” algorithm, the “LS-SOCP2” algorithm, the “SOCP2” method, the “DEOR2” method and the “DEOR-fast2” method. In addition, the Cramer–Rao lower bound (CRLB) is also provided in both conditions. CRLB is the best effect that can estimate parameters by using the existing information. The closer to CRLB, the better the performance of the algorithm.

All RSS measurements are generated by Equation (1). The measurement noise is based on normal distribution. Anchors are randomly generated in a square area with a side length of 100 m. In order to avoid the influence of the special distribution of anchors, the position of anchors is reset in each simulation. The settings of reference distance, path loss, reference measured value, simulation times and other variables are given in Table 2. The root mean square error (RMSE) is used to evaluate the performance of the algorithm. The definition of RMSE is $\sqrt{\frac{1}{M_{c}}\sum_{i=1}^{Mc}{∥x_{i}-{\hat{x}}_{i}∥}^{2}}$, where $x_{i}$ is the position of the target, ${\hat{x}}_{i}$ is the estimated position of the target and Mc is the simulation times.

The variables of $\sigma_{n_{i}}$ and *N* are given in Table 3.

### 4.1. $L_{0}$ Is Known

Average running times of the eight algorithms are listed in Table 4. From Table 4, we can know that the average running time of the proposed algorithm in this paper is 0.58 s, which means that while improving the accuracy, the running times do not increase. The bias of the “ML-SDP1” method is 0.0567 m, according to the simulation results.

First, we test the relationship between the RMSE of the estimator of the target and $\sigma_{n_{i}}$ (the variance of the measurement noise). Nine anchors are randomly distributed, and the target is randomly generated. σ changes from 1 to 6 dBm.

**Figure 2:**
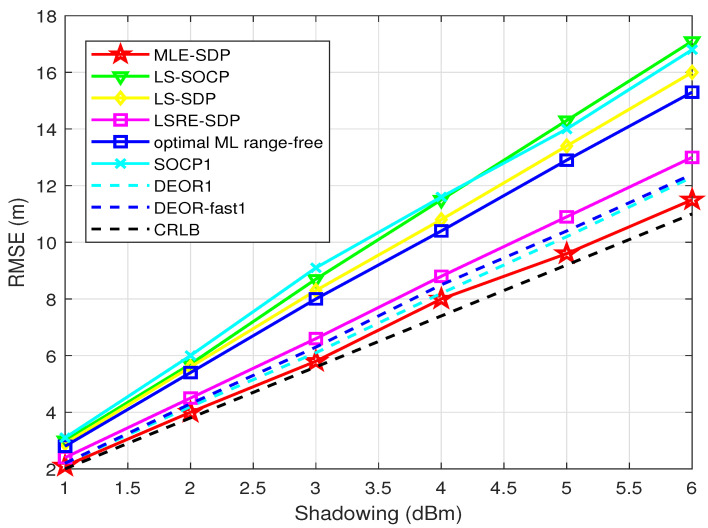
When $L_{0}$ is known, this is the relationship between RMSE and $\sigma_{n_{i}}$.

Figure 2 shows the comparison between the proposed algorithm and other algorithms. The RMSE of all algorithms increases with the increase in the measurement noise $\sigma_{n_{i}}$, which indicates that the larger the noise, the larger the deviation of the final estimator. The proposed algorithm “MLE-SDP” in this paper has lower RMSE than the other seven algorithms and is the closest to the CRLB. This also shows that the proposed algorithm has a significant advantage in accuracy.

**Figure 3:**
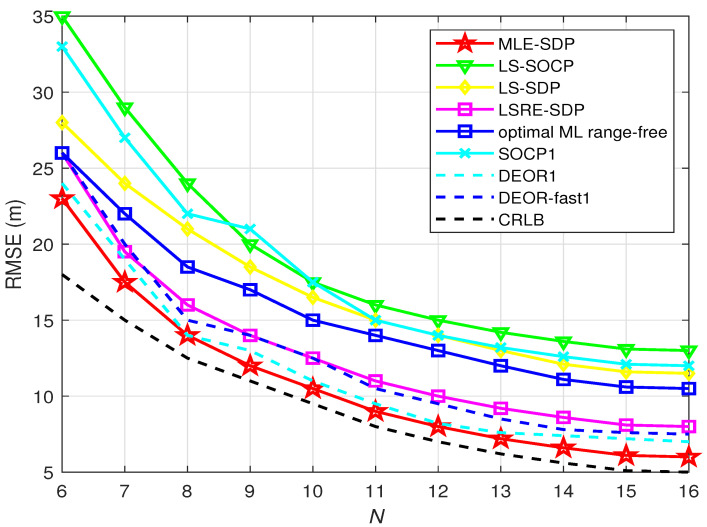
When $L_{0}$ is known, this is the relationship between RMSE and *N*.

Figure 3 shows the RMSE of the estimator of the target versus the number of anchors. It is obvious that the RMSE of all algorithms reduces while the number of anchors increases. $\sigma_{n_{i}}$ is set to be 4 dBm.

As shown in Figure 3, compared with the other seven algorithms, the RMSE of the proposed algorithm is lower whether the number of anchor nodes is large or small. It is very close to the CRLB when the number of anchors is larger than 8. That means it has better performance when the number of anchor nodes is larger than 8.

**Figure 4:**
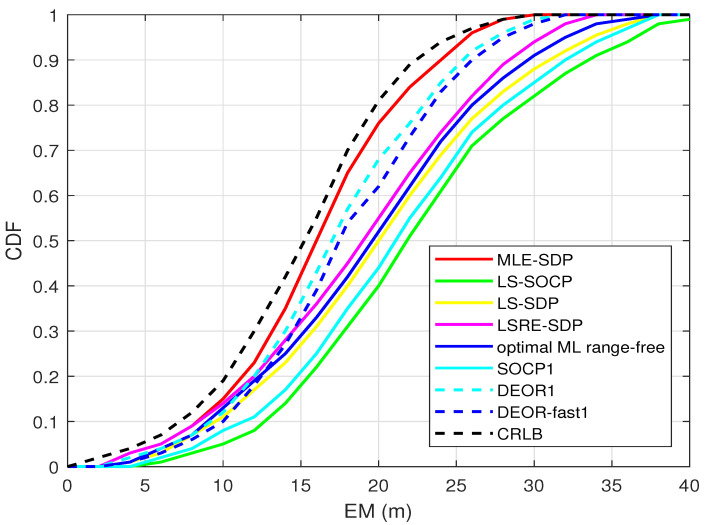
When $L_{0}$ is known, this is the CDF.

Figure 4 shows the cumulative distribution function (CDF) versus the mean error (EM). The faster the CDF curve rises, the better the performance is. Nine anchors are randomly distributed, and the target is randomly generated. The standard deviation of RSS measurement noise is set as σ = 4 dBm.

As shown in Figure 4, the CDF of the proposed algorithm is higher than the other seven algorithms. Moreover, it is the closest to the CRLB, which means it has higher accuracy.

### 4.2. $L_{0}$ Is Unknown

The average running times of the six algorithms which $L_{0}$ are unknown are listed in Table 5. From Table 5, we can know that the running times of the proposed algorithm “MLS-SDP2” in this paper are almost twice the running times of “MLS-SDP1”. This is due to the increase in constraints and unknown variables. The bias of the “ML-SDP2” method is 0.0768 m, according to the simulation results.

First, we test the relationship between the RMSE of the estimator of the target and $\sigma_{n_{i}}$. Nine anchor nodes are randomly distributed, and the target is randomly generated. σ change from 1 to 6.

**Figure 5:**
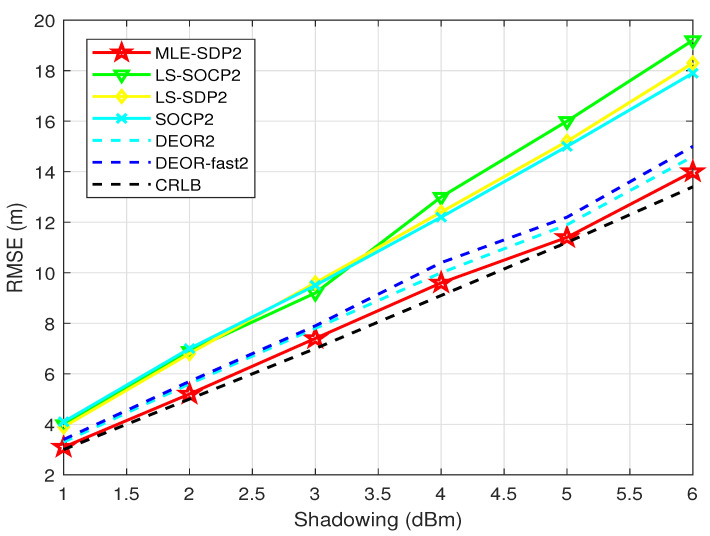
When $L_{0}$ is unknown, this is the relationship between RMSE and $\sigma_{n_{i}}$.

Figure 5 shows the comparison between the proposed algorithm and other algorithms. The RMSE of all algorithms increases with the increase in the measurement noise $\sigma_{n_{i}}$, which indicates that the larger the noise, the larger the deviation of the final estimator. The proposed algorithm “MLE-SDP2” in this paper has a lower RMSE than the other five algorithms and is the closest to the CRLB. This also shows that the proposed algorithm has a significant advantage in accuracy.

**Figure 6:**
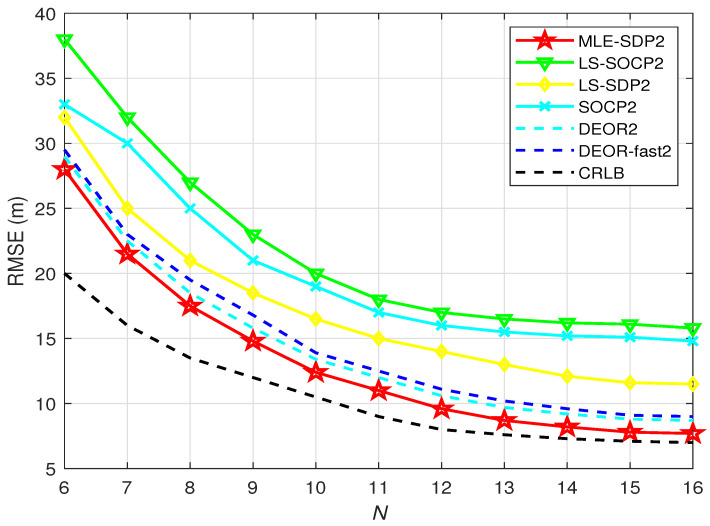
When $L_{0}$ is unknown, the relationship between RMSE versus *N*.

Figure 6 shows the RMSE versus the number of anchors when $L_{0}$ is unknown. It is obvious that the RMSE of all algorithms reduces while the number of anchors increase. The standard deviation of RSS measurement noise is set as $\sigma_{n_{i}}$ = 4 dBm.

As shown in Figure 6, compared with the other five algorithms, the RMSE of the proposed algorithm is lower whether the number of anchors is large or small. It is very close to the CRLB when the number of anchor nodes is larger than 10. That means the proposed algorithm has a better performance when the anchor nodes are more than 10.

**Figure 7:**
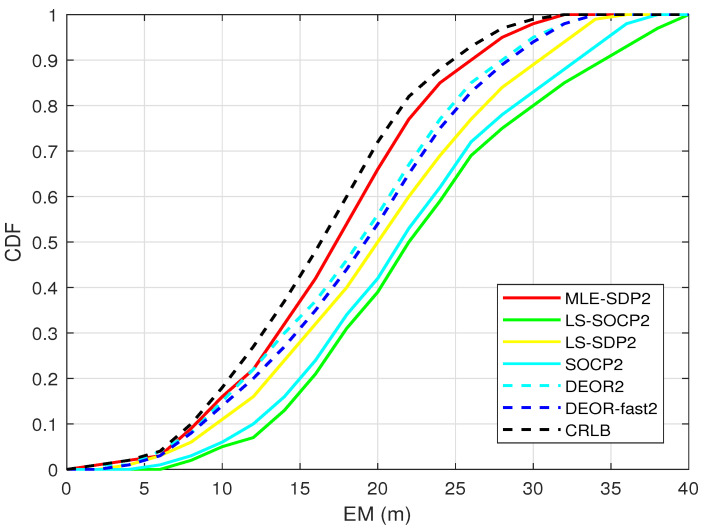
When $L_{0}$ is unknown, this is the CDF.

Figure 7 shows the relationship between the cumulative distribution function (CDF) and the mean error (EM). The faster the CDF curve rises, the better the performance is. Nine anchors are randomly distributed, and the target is randomly generated. The standard deviation of RSS measurement noise is set as σ = 4 dBm.

As shown in Figure 7, the CDF of the proposed algorithm is higher than the other two algorithms. In addition, it is the closest to the CRLB, which means it has higher accuracy.

## 5. Experiment

**Figure 8:**
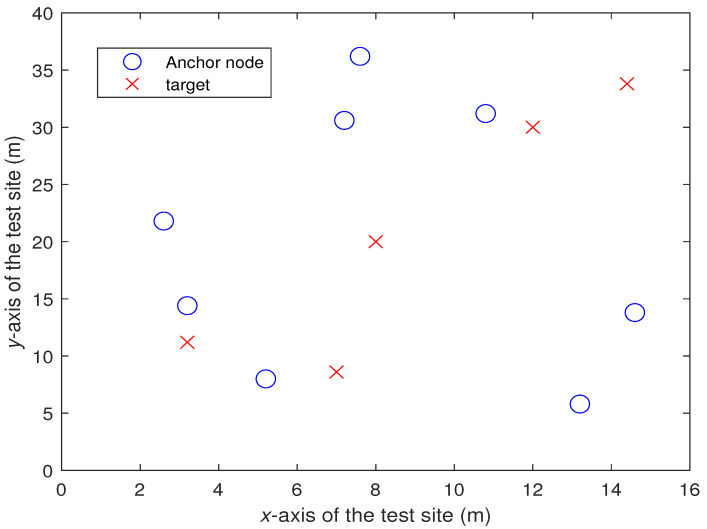
Distribution of the anchor nodes and the targets.

This section shows the effect of the proposed algorithm “MLE-SDP2” in the actual test. The actual test was carried out in the rectangular laboratory of 40 m long and 16 m wide. We put 8 anchor nodes and 5 target to test. The location of the anchors is fixed each time, while the location of the target is randomly placed, as shown in Figure 8. In Figure 8, the abscissa and ordinate represent the width and length of the test site, respectively. The units are both m.

**Figure 9:**
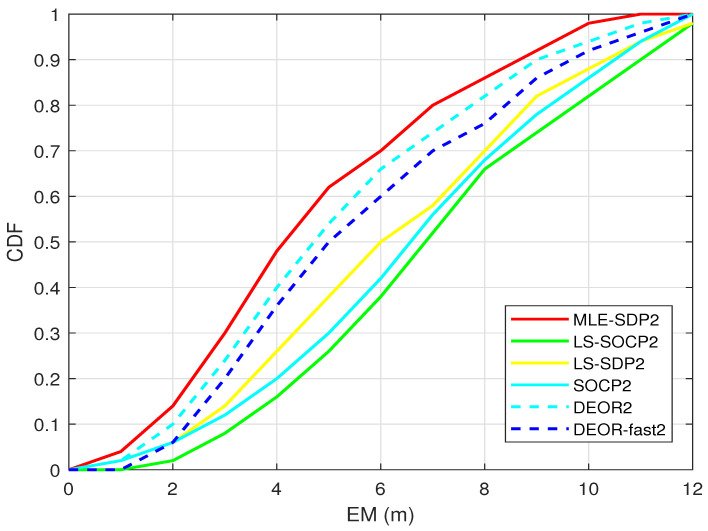
The CDF of the experiments.

For each target, we test 100 sets of data; therefore, we collect a total of 5100 sets of data. Figure 9 shows the relationship between the cumulative distribution function (CDF) and the mean error (EM) in this experiment.

As shown in Figure 9, the CDF of the proposed algorithm is higher than the other five algorithms. This proves the good performance of the proposed algorithm.

## 6. Conclusions

In this paper, a new algorithm based on maximum likelihood criterion for unknown point location using semi-definite programming estimation is studied. The estimators are proposed for the cases where $L_{0}$ is known and unknown. Through MATLAB simulation, the advantage of the proposed methods “MLE-SDP” and “MLE-SDP2” in calculation accuracy is confirmed. The experiments show the practicability of this algorithm. A non-line of sight (NLOS) error is the main component of measurement error. In the future work, we will model the NLOS error so that the algorithm can be better applied to practice.

## Figures and Tables

**Table 1:** Symbols and notations.

Symbol	Explanation
$L_{i}$	The RSS measurements
$d_{0}$	The reference distance
$L_{0}$	The RSS measurements when $d_{0}=1$
γ	The pass-loss exponent
x	The position of the target node
$s_{i}$	The position of the *i*-th anchor node
*N*	The number of anchor nodes
$d_{i}$	The range between the target node and the *i*-th anchor node
$n_{i}$	The measurement noise between the target node and the *i*-th anchor node
σ	The standard deviation of measurement noises

**Table 2:** Simulation parameter settings.

Symbol	Describe	Value
$d_{0}$	reference distance	1 m
γ	path loss	4
$L_{0}$	reference measured value	−10 dBm
Mc	simulation times	5000

**Table 3:** Variables in Figure 2, Figure 3, Figure 4, Figure 5, Figure 6 and Figure 7.

	$\sigma_{n_{i}}$	*N*
Figure 2	1–6 dBm	9
Figure 3	4 dBm	6–16
Figure 4	4 dBm	9
Figure 5	1–6 dBm	9
Figure 6	4 dBm	6–16
Figure 7	4 dBm	9

**Table 4:** Average running times of $L_{0}$ known situation.

Method	Describe	Running Times (s)
MLE-SDP	The “ML-SDP” algorithm in this paper	0.58
LS-SDP	The “LS-SDP” algorithm in [30]	0.85
LS-SOCP	The “LS-SOCP” algorithm in [31]	1.16
LSRE-SOCP	The “LSRE-SOCP” algorithm in [32]	2.53
optimal-ML	The “optimal ML range-free” algorithm in [37]	3.58
SOCP1	The “SOCP1” algorithm in [35]	2.87
DEOR1	The “DEOR” algorithm in [38]	0.45
DEOR-fast1	The “DEOR-fast” algorithm in [38]	0.23

**Table 5:** Average running times of $L_{0}$ unknown situation.

Method	Describe	Running Times (s)
MLE-SDP2	The “ML-SDP2” algorithm in this paper	1.13
LS-SDP2	The “LS-SDP2” algorithm in [30]	1.65
LS-SOCP2	The “LS-SOCP2” algorithm in [31]	2.28
SOCP2	The “SOCP2” algorithm in [35]	5.43
DEOR2	The “DEOR” algorithm in [38]	0.63
DEOR-fast2	The “DEOR-fast” algorithm in [38]	0.32

## Data Availability

Not applicable.

## Abbreviations

The following abbreviations are used in this manuscript:

WSNs	Wireless sensor networks
AOA	Angle of arrival
TOA	Time of arrival
TDOA	Time difference of arrival
RSS	Received signal strength
SDP	Semi-definite programming
SOCP	Second-order cone programming
RMSE	Root mean square error
CDF	Cumulative distribution function
CM	Mean error
